# Atroposelective antibodies as a designed protein scaffold for artificial metalloenzymes

**DOI:** 10.1038/s41598-019-49844-0

**Published:** 2019-09-19

**Authors:** Takuma Adachi, Akira Harada, Hiroyasu Yamaguchi

**Affiliations:** 1 Department of Macromolecular Science, Graduate School of Science, Osaka University, Toyonaka, Osaka 560-0043 Japan; 2 The Institute of Scientific and Industrial Research, Osaka University, Ibaraki, 567-0047 Japan

**Keywords:** Biocatalysis, Biocatalysis, Supramolecular chemistry, Supramolecular chemistry

## Abstract

Design and engineering of protein scaffolds are crucial to create artificial metalloenzymes. Herein we report the first example of C-C bond formation catalyzed by artificial metalloenzymes, which consist of monoclonal antibodies (mAbs) and *C*_2_ symmetric metal catalysts. Prepared as a tailored protein scaffold for a binaphthyl derivative (BN), mAbs bind metal catalysts bearing a 1,1′-bi-isoquinoline (BIQ) ligand to yield artificial metalloenzymes. These artificial metalloenzymes catalyze the Friedel-Crafts alkylation reaction. In the presence of mAb R44E1, the reaction proceeds with 88% ee. The reaction catalyzed by Cu-catalyst incorporated into the binding site of mAb R44E1 is found to show excellent enantioselectivity with 99% ee. The protein environment also enables the use of BIQ-based catalysts as asymmetric catalysts for the first time.

## Introduction

Artificial metalloenzymes, which consist of transition metal catalysts and biomolecular scaffolds, offer new reactivities or selectivities that are not observed in nature or synthetic catalysts^1–6^. One way to create artificial metalloenzymes is to engineer natural enzymes to yield non-natural reactivities that combine the attractive features of both the metal catalyst and bio-molecular scaffold^7–14^. Another strategy is to incorporate synthetic metal catalysts into bimolecular scaffolds by covalent^15–20^, dative^21,22^ or supramolecular interaction^23–29^. This strategy has also been applied to non-enzymatic proteins or DNAs. However, there are a limited number of existing protein scaffolds that can be used to implement the aforementioned design strategies. Therefore, the choice and engineering of biomolecular scaffolds along with the synthetic optimization of cofactors or conjugation technologies are also routinely required. An alternative to these strategies is the *de novo* creation of tailored protein scaffolds with immunological optimization to provide a chiral environment around the metal complex. Monoclonal antibodies (mAbs), which are chemically homogeneous antibodies^30^, have received much attention as designable protein scaffolds for artificial metalloenzymes^31–39^.

**Figure 1:**
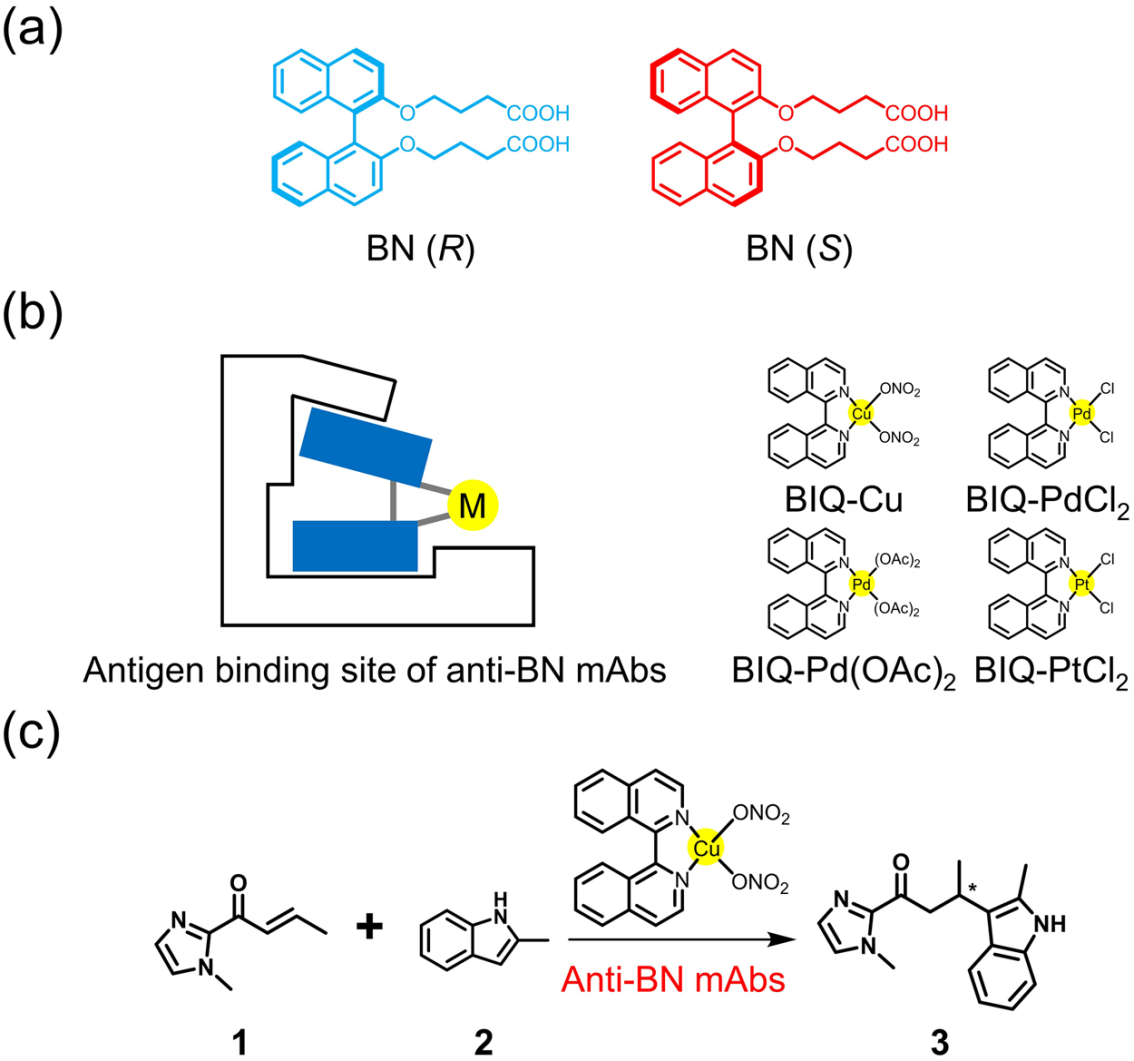
Design strategy for artificial metalloenzymes based on atroposelective antibodies. Atroposelective antibodies generated against a structurally simple binaphthyl derivative (BN) (**a**) are used to accommodate various BIQ-based metal catalysts (**b**). Catalytic asymmetric Friedel-Crafts alkylation reaction is realized by just adding atroposelective antibodies to the mixture of BIQ-Cu and substrates (**c**).

Our research focuses on the binaphthyl group as a target molecule to complex with mAbs. 2,2′-Bis(diphenylphosphino)-1,1′-binaphthyl (BINAP) has a unique structure where two phosphine atoms located at the 2,2′ position of the binaphthyl groups play a key role in stabilizing the unique chiral structure and coordination behavior^40,41^. Due to atropisomeric instability, structurally similar ligands with binaphthyls such as 1,1′-bi-isoquinoline (BIQ) have not been used in asymmetric catalysis^42^. We expect that supramolecular complexation of BIQ-based metal catalysts with mAbs will enhance the diversity of available asymmetric catalysts. Recently, we revealed that mAbs prepared by immunization with *R*-and *S*-4,4′-([1,1′-binaphthalene]-2,2′-diylbis(oxy))dibutanoic acid (BN (*R*) and BN (*S*) in Fig. 1a) or racemic BN precisely recognize the axial chirality of BN^43,44^. Hence, we defined the anti-BN mAbs as an atroposelective antibody. The chiral recognition ability has been applied to operationally simple and rapid chiral separation and chiral sensing systems^43,45^.

Herein we report a design strategy for artificial metalloenzymes based on supramolecular complexation of BIQ-based metal catalysts with atroposelective antibodies generated against a structurally simple hapten (Fig. 1b). The resulting artificial metalloenzymes with BIQ-Cu as a cofactor in the binding sites of mAbs catalyze the Friedel-Crafts alkylation reaction with up to 88% ee (Fig. 1c). This result implies that the reaction catalyzed by Cu-catalyst incorporated into the binding site of mAb R44E1 shows enantioselectivity with 99% ee.

## Results and Discussion

**Figure 2:**
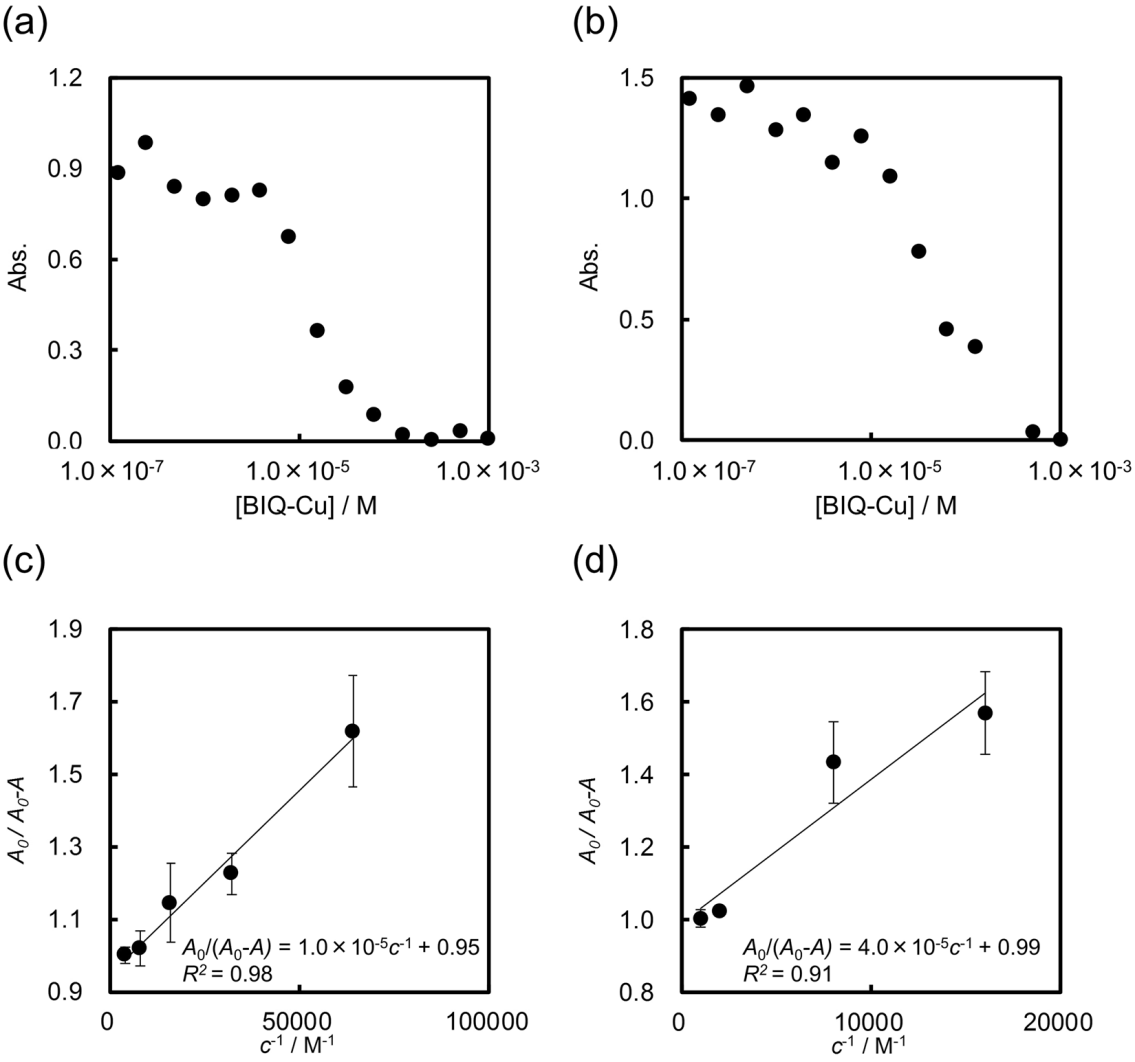
Competitive ELISA of mAb R44E1 (**a**) and mAb S1E11 (**b**) for BIQ-Cu and corresponding Klotz plots (**c**) and (**d**), respectively.

We prepared four BIQ-based metal complexes: BIQ-Cu, BIQ-PdCl_2_, BIQ-Pd(OAc)_2,_ and BIQ-PtCl_2_. The binding affinity of mAbs to the four BIQ-based metal complexes was evaluated by competitive ELISA. Both anti-BN (*R*) mAb R44E1 and anti-BN (*S*) mAb S1E11 bind all metal catalysts with *K*_d_ values ranging from 10^**−**4^ M to 10^**−**5^ M (Table 1, Figs 2 and S1–S7). Supramolecular complexes of atroposelective antibodies with BIQ-based matal complexes are successfully developed. Additionally, mAbs R44E1 and S1E11 show the highest affinity toward BIQ-Cu (Fig. 2). Especially, mAb R44E1 has a higher affinity for BIQ-Cu compared to mAb S1E11 (*K*_d_ = 1.0 × 10^**−**5^ M). Given the higher affinity of mAbs for a metal complex provides the higher effect of the binding of mAbs, we selected complexes of mAbs with BIQ-Cu for further investigations.

**Table 1:** Dissociation constants (*K*_d_) of the complexes between mAbs and BIQ-based metal complexes, **1**, **2**, or **3**.

mAb	*K*_d_/M						
	BIQ-Cu	BIQ-PdCl_2_	BIQ-Pd(OAc)_2_	BIQ-PtCl_2_	1	2	3
R44E1	1.0 × 10^−5^	~10^−5^	4.9 × 10^−5^	1.6 × 10^−4^	>1.0 × 10^−3^	4.8 × 10^−3^	4.8 × 10^−5^
S1E11	4.0 × 10^−5^	~10^−5^	~10^−5^	2.3 × 10^−4^	>1.0 × 10^−3^	6.5 × 10^−3^	>5.0 × 10^−4^

The Friedel-Crafts alkylation reaction was carried out by mixing atroposelective antibodies (50 μM) with BIQ-Cu (50 μM) in 20 mM MOPS buffer (pH 6.5) containing 150 mM NaCl followed by the addition of substrates (1.0 mM). Under these conditions, the molar ratio of antigen binding sites to BIQ-Cu is two to one. The reactions were carried out at 4 °C for 72 h. The product was analyzed by chiral HPLC. Although BIQ-Cu affords racemic **3** with 6% yield (Table 2, Entry 1), the supramolecular complex of mAb S1E11 with BIQ-Cu yields **3** in 2% yield, 65% ee (Table 2, Entry 2). The complexes of mAb R44E1 and BIQ-Cu catalyze the reaction with 10% yield, 88% ee (Table 2, Entry 3). These results suggest that precisely designed second coordination spheres control the reactivity and enantioselectivity of the asymmetric catalysis. Interestingly, both of mAb R44E1 and mAb S1E11 give (+)-**3**, though the binding selectivities of these mAbs are opposite. Our recent study demonstrates that these mAbs recognize the axial chirality of BN by binding the crossing moiety of two naphthyl rings (Fig. S8)^44^. The binding pocket of mAb R44E1 prepared for BN (*R*) is thought to induce twisted conformation on the bound BIQ-Cu. The induced chirality is considered to increase in the yield and the enantioselectivity of the catalytic reaction. In contrast, the yield in the presence of mAb S1E11 is lower compared to that of BIQ-Cu alone. The affinity of mAb S1E11 for BIQ-Cu is also lower than that of mAb R44E1. This suggests that the binding modes of the two mAbs are different to provide different environments around the bound BIQ-Cu. The microenvironment formed by mAb S1E11 is suggested to regulate the accessibility of substrates to the reaction center to give the same enantiomer of product **3** that produced by R44E1 ⊃ BIQ-Cu. Although anti-porphyrin mAb 2B6^46,47^ has an unexpected affinity for BIQ-Cu (Fig. S7, *K*_d_ = 7.1 × 10^−5^ M), presumably due to hydrophobic interactions, catalytic reaction in the presence of BIQ-Cu and 2B6 gives racemic **3** (Table 2, Entry 4). In another control experiment, bovine serum albumin (BSA) does not affect the reactivity and enantioselectivity of the catalytic reaction (Table 2, Entry 5). These two control experiments indicate that the protein scaffolds must be optimized immunologically to prepare enantioselective artificial metalloenzymes.

**Table 2:** Friedel-Crafts alkylation reactions catalyzed by artificial metalloenzymes based on atroposelective antibodies.

Entry	Catalyst	Yield/%^*a*^	ee/%^*b*^
1	BIQ-Cu	6	0
2	S1E11 + BIQ-Cu	2	65
3	R44E1 + BIQ-Cu	10	88
	R44E1 ⊃ BIQ-Cu (85%^*c*^)	9	99
	BIQ-Cu (15%^*c*^)	1	0
4	2B6 + BIQ-Cu	17	2
5	BSA + BIQ-Cu	8	3

Typical reaction conditions: 1.0 mM of substrate **1** and **2**, 50 μM of mAb (5.0%), 50 μM of BIQ-Cu (5.0%) in 20 mM MOPS buffer (pH 6.5), 150 mM NaCl at 4 °C for 72 h. Conditions for HPLC analysis: Daicel ChiralPak AD-H, hexane/2-propanol (90/10), 1.0 mL/min, 40 °C, UV and CD detector at 275 nm and 280 nm. ^*a*^Yields were determined by HPLC using 2-phenylquinoline as an internal standard. ^*b*^ee of (+) isomer. (−) and (+) isomers of **3** are defined based on the HPLC analysis with UV and CD detector. ^*c*^Based on the *K*_d_ of the complex of mAb R44E1 with BIQ-Cu, 85% of BIQ-Cu is bound by mAb R44E1 under the reaction conditions.

To further analyze the effects of protein environments formed by mAbs on the catalytic reaction, the affinity of mAbs for substrates **1**, **2**, and product **3** of the Friedel-Crafts alkylation reaction was evaluated by competitive ELISA. mAbs do not bind **1**. In contrast, they have a weak affinity for **2**, even though the interaction with **2** is not immunologically installed (Table 1). Apparently, the weak affinity appears to be non-specific binding of hydrophobic indole derivative **2**. However, the other atroposelective antibody does not bind it at all. Therefore, the interaction of mAbs to **2** is attributed to the structural nature of each mAb. Interestingly, mAb R44E1 also binds product **3** with a higher affinity than that to **2**. In contrast, mAbs S1E11 and **3** do not interact. These difference in affinity is derived from the structural difference of protein environment between two mAbs. Even if the mAbs are elicited for the same hapten, the structure, binding and catalytic behavior is different. This is unique feature of mAbs as a protein scaffold for Friedel-Crafts-ase^27,48–52^.

The catalytic reaction occurred inside the binding site of mAb R44E1-based artificial metalloenzyme is analyzed in detail. The affinity of mAb R44E1 for **2** (*K*_d_ = 4.8 × 10^−3^ M) seems to contribute to the increase in yield compared to BIQ-Cu. The affinities of mAb R44E1 for product **3** may provide the stabilization of an enantiomer of **3** inside the binding pockets to increase the enantioselectivity. The mAb R44E1 binds 85% of BIQ-Cu under the reaction condition (*K*_d_ = 1.0 × 10^−5^ M). The reaction catalyzed by BIQ-Cu incorporated into the binding site of mAb R44E1 (R44E1 ⊃ BIQ-Cu) is found to proceed with excellent enantioselectivity (99% ee) when the contribution of unbound BIQ-Cu is excluded. Importantly, the immunologically optimized atroposelective antibodies as a protein scaffold realize stereocontrol of the catalytic reaction.

In summary, a novel design strategy for artificial metalloenzymes is developed by introducing BIQ-based metal catalysts into the binding sites of atroposelective antibodies. The atroposelective antibodies for BN bind to BIQ-based metal catalysts. Artificial metalloenzymes bearing BIQ-Cu as a cofactor catalyze Friedel-Crafts alkylation of **1** with **2** with high ee. The specific protein environment formed by mAbs controls the enantioselectivity of the catalytic reaction. Especially, the enantioselectivity of the catalytic reaction caused by the binding of mAb R44E1 to BIQ-Cu is excellent. Importantly, this is the first example of C-C bond formation catalyzed by artificial metalloenzymes based on mAbs. In addition, a BIQ-based catalyst is used as an asymmetric catalyst for the first time by supramolecular complexation with mAbs. The design strategy for artificial metalloenzymes developed herein accepts various metal catalysts with BIQ or binaphthyl-based ligand, which will allow more precise control of stereochemistry or a range of catalytic reaction, including abiological reactions.

## Methods

General procedure for Friedel-Crafts alkylation reaction: Catalytic reaction was performed in 150 μL total volume containing 1.0 mM of substrates, 50 μM of BIQ-Cu (5.0%) and 50 μM of mAb (5.0%) in 20 mM MOPS buffer (pH 6.5), 150 mM NaCl. The reaction mixture was incubated at 4 °C for 72 h followed by addition of 2-phenyl quinolone as an internal standard for HPLC analysis. The mixture was extracted with diethyl ether (300 μL × 3) and the combined organic layer was dried over Na_2_SO_4_ and evaporated under reduced pressure to give the product. The yield and ee were determined by chiral HPLC analysis.

## Supplementary information

Supporting Information
