# Supplementary material for: Atroposelective antibodies as a designed protein scaffold for artificial metalloenzymes
Source: Sci Rep. 2019 Sep 19;9:13551. doi: 10.1038/s41598-019-49844-0 (PMC6753118; doi:10.1038/s41598-019-49844-0)
Supplement: Supplementary file 1 — Supporting Information [file 41598_2019_49844_MOESM1_ESM.docx]

**Supporting Information**

**Atroposelective antibodies as a designed protein scaffold for artificial metalloenzymes**

Takuma Adachi^1^, Akira Harada^2^* and Hiroyasu Yamaguchi^1^*

^1^*Department of Macromolecular Science, Graduate School of Science, Osaka University, Toyonaka, Osaka, 560-0043 (Japan)*

^2^The Institute of Scientific and Industrial Research, Osaka University, Ibaraki 567-0047 (Japan)

**Table of Contents**

1. Materials and measurements---------------------------------------------------------------------------------2

2. Preparation of 1,1’-bi-isoquinoline (BIQ)-based metal complexes------------------------------------ 3

3. Preparation of substrates and standard sample of Friedel-Crafts alkylation reaction product------5

4. Dissociation constants (*K*_d_) of the complexes between monoclonal antibodies (mAbs) and metal complexes------------------------------------------------------------------------------------------------------7

5. Docked structures of mAbs with BN enantiomers-------------------------------------------------------10

6. General procedure for Friedel-Crafts alkylation reaction-----------------------------------------------10

7. Dissociation constants (*K*_d_) of the complexes between mAbs and substrates or product of Friedel-Crafts alkylation reaction-----------------------------------------------------------------------------------11

8. NMR spectra--------------------------------------------------------------------------------------------------14

9. Chiral HPLC charts------------------------------------------------------------------------------------------17

10. References---------------------------------------------------------------------------------------------------19

**1. Materials and measurements**

Materials

KH_2_PO_4_, Na_2_B_4_O_7_·10H_2_O, MgSO_4_, Na_2_SO_4_, NaHCO_3,_ NaCl, _,_ CuNO_3_・3H_2_O, Zn, NH_3_ aq., and *p*-nitrophenyl phosphate sodium salt were purchased from Nakalai Tesque, Inc. 1-Chloroisoquinoline, tetramethylammonium iodide (Et_4_NI), 1-methyl-1H-imidazole, crotonic acid, 2-methyl-1H-indole, PdCl_2_(CH_3_CN)_2,_ Pd(OAc)_2_, 2-phenyl quinolone were purchased from Tokyo Kasei Inc. Dibromobis(triphenylphosphine)nickel(II) (NiBr_2_(PPh_3_)) was purchased from Sigma Aldrich Inc. K_2_PtCl_4_ was purchased from Wako Pure Chemical Industries, Ltd. *n*-BuLi was purchased from Kanto Chemical Co., Inc. BSA was purchased from Medical & Biological Laboratories Co. Ltd. Alkaline phosphatase labeled anti-mouse IgG were obtained from Sigma Aldrich Co. Reagents were used without further purification. The buffer compositions are as follows. 0.1M PBB: KH_2_PO_4_ 3.97 g, Na_2_B_4_O_7_·10H_2_O 26.3 g/ H_2_O 1 L. Washing buffer: 10 mM PBS containing 20 mM NaN_3_ and 10 mM of Tween 20. Substrate buffer was prepared by dissolving 1 M di-ethanolamine, 3 mM sodium azide, and 1 mM MgCl in water and pH was adjusted to 9.8 by hydrochloric acid solution.

Measurements

The ^1^H NMR and ^13^C NMR spectra were recorded at 500 MHz with a JEOL JNM-ECA 500 NMR spectrometer. The absorption spectra were measured with a JASCO V-650 and a Shimadzu UV-2500 PC spectrometer at room temperature. Circular dichroism spectra were recorded with a JASCO J820 spectrometer. Absorbance at 405 nm for ELISA was measured by an iMark microplate absorbance reader (Bio-Rad). The chiral HPLC analyses were carried out using JASCO HPLC system equipped with a HPLC pump (PU-2080), an UV-Vis detector (UV-2075), a CD detector (CD-4095), and a Daicel ChiralPak AD-H (4.6×250 nm). Condition for HPLC analyses is hexane/2-propanol (90/10), 40 °C, 1.0 mL/min, UV and CD detection at 275 and 280 nm.

**2. Preparation of 1,1’-bi-isoquinoline (BIQ)-based metal complexes**

**BIQ**

**Scheme S1.** Synthesis of 1,1’-bi-isoquinoline (BIQ).

THF (20.0 mL) was added to NiBr_2_(PPh_3_) (2.27 g, 3.04 mmol), Zn (1.03 g. 25.8 mmol), Et_4_NI (2.71 g, 10.5 mmol). The reaction mixture was stirred for 30 minutes under nitrogen atmosphere and then a solution of 1-chloroisoquinoline (1.05 g, 6.37 mmol) in THF (10.0 mL) was added. The resulting mixture was refluxed for 23 h and solvent was removed under reduced pressure. CH_2_Cl_2_ (50 mL) was added to black solid and filtered. 2M NH_3_ aq. (60 mL) was added to the filtrate and was extracted with CH_2_Cl_2_ (50 mL×3). The combined organic phase was washed with 2 M NH_3_ aq. (30 mL×2), water (30 mL×1) and sat. NaCl aq. (30 mL×1) and dried over MgSO_4_. The solvent was removed under reduced pressure. Purification by column chromatography (SiO_2_, hexane/EtOAc (95/5), CHCl_3_/MeOH (96/4)) gave BIQ as a yellow solid,^1^ (603 mg, 74%). ^1^HNMR (500 MHz, CDCl_3_) δ 7.48 (td, *J*=1.4, 6.0 Hz, 1H), 7.70 (m, 1H), 7.75 (d, *J*=7.4 Hz, 1H), 7.82 (d, *J*=5.7 Hz, 1H), 7.95 (d, *J*=8.3 Hz, 1H), 8.71 (d, *J*=5.7 Hz, 1H). ^13^CNMR (125 MHz, CDCl_3_) δ 121.18, 127.98, 130.48, 136.99, 142.10, 158.28. ESI-TOF-MS: *m*/*z* Calcd. for C_18_H_12_N_2_Na ([M+Na]^+^) 279.09; Found 279.10

**BIQ-Cu**

**Scheme S2.** Synthesis of BIQ-Cu.

To a solution of BIQ (106 mg, 0.414 mmol) in ethanol (10 mL) was added a solution of CuNO_3_・3H_2_O (109 mg, 0.452 mmol) in ethanol (5.0 mL). The resulting mixture was stirred for 2 h at room temperature under air. The suspension was filtered and the solid was washed with cold ethanol. Dark green solid,^2^ 108 mg, 59%. Elemental analysis Calcd. for C_18_H_12_CuN_4_O_6_: C 48.71, H 2.73, Cu 14.32, N 12.62, O 21.63N 1.00; found C 47.69, H 3.17, N 12.03

**BIQ-PdCl_2_**

**Scheme S3.** Preparation of BIQ-PdCl_2_.

To a solution of BIQ (102 mg, 0.398 mmol) in CH_2_Cl_2_ (1.0 mL) was added a suspension of PdCl_2_(CH_3_CN)_2_ (104 mg, 0.402 mmol) in CH_2_Cl_2_ (8.0 mL). The resulting mixture was stirred for 2 h at room temperature under air. Hexane (20.0 mL) was added, and the precipitate was filtered and dried under reduced pressure. Dark green solid,^3,4^ 166 mg, 96%. ^1^HNMR (500 MHz, DMSO-*d*_6_) δ 7.72 (t, *J*=7.5, 15.5 Hz, 1H), 7.85 (d, *J*=8.5 Hz, 1H), 8.04 (t, ­*J*=7.0, 15.0 Hz, 1H), 8.29 (d, ­*J*=8.0 Hz, 1H), 8.43 (d, *J*=6.0 Hz, 1H), 9.15 (d, *J*=6.5Hz, 1H). ^13^CNMR (500 MHz, DMSO-*d*_6_) δ 125.42, 126.89, 127.51, 128.25, 129.04, 133.37, 137.91, 141.00, 157.90

**BIQ-Pd(OAc)_2_**

**Scheme S4.** Preparation of BIQ-Pd(OAc)_2_.

To a solution of BIQ (102 mg, 0.398 mmol) in CH_2_Cl_2_ (5.0 mL) was added a suspension of Pd(OAc)_2_ (92.5 mg, 0.413 mmol) in CH_2_Cl_2_ (10.0 mL). The resulting mixture was stirred for 2 h at room temperature under air. Hexane (60.0 mL) was added, and the precipitate was filtered and dried under reduced pressure. Brown solid, 72.4 mg, 38%. ^1^HNMR (500 MHz, DMSO-*d*_6_) δ 7.74 (m, 1H), 7.89 (d, *J*=9.0 Hz, 1H), 8.03 (t, ­*J*=7.5, 15 Hz, 1H), 8.18 (d, ­*J*=6.0 Hz, 1H), 8.30 (d, *J*=8.5 Hz, 1H), 8.41 (d, *J*=10 Hz, 1H). ^13^CNMR (500 MHz, DMSO-*d*_6_) δ 23.48, 125.76, 126.61, 127.62, 127.84, 129.14, 137.94, 140.66, 156.70, 175.73, 180.26

**BIQ-PtCl_2_**

**Scheme S5.** Preparation of BIQ-PtCl_2_.

BIQ (280 mg, 1.09 mmol) and K_2_PtCl_4_ (445 mg, 1.07 mmol) were refluxed in water (80 mL) for 2 h. The reaction mixture was cooled to 0 °C. The solid precipitate was filtered and washed with EtOH, 1M HCl and water. Orange solid,^5^ 460 mg (82%). Elemental analysis Calcd. for C_18_H_12_Cl_2_N_2_Pt: C 41.39, H 2.32, Cl 13.57, N 5.36, Pt 37.35; found C 41.63, H 2.49, N 5.45

**3. Preparation of substrates and standard sample of Friedel-Crafts alkylation reaction product**

**Substrate 1**

**Scheme S6.** Preparation of **1**.

1-Methyl-1H-imidazole (**4**, 1.50 mL, 19.0 mmol) and THF (25.0 mL) were added to an oven-dried schlenk tube under nitrogen atmosphere. The solution was cooled to −78 °C and *n*-BuLi (1.5M in hexane, 17.0 mL, 25.5 mmol) was added dropwise over 25 min. The mixture was warmed to room temperature and stirred for 20 min, then cooled back to −78 °C. Crotonic acid (**5**, 793 mg, 9.2 mmol) in THF (10.0 mL) was added dropwise over 5 min. The resulting solution was stirred at −78 °C for 20 min, then warmed to room temperature and stirred for additional 2 h. The reaction was quenched with sat NaHCO_3_ solution (50 mL) and the aqueous phase was extracted with EtOAc (40 mL×3). The combined organic phase was washed with brine (30 mL×2), dried over MgSO_4_, and the solvent was removed under reduced pressure. Purification by column chromatography (SiO_2_, hexane/EtOAc (72/28) gave 1-(1-methyl-1H-imidazol-2-yl)but-2-en-1-one (**1**) as a yellow liquid,^6^ (291 mg, 21%). ^1^HNMR (500 MHz, CDCl_3_) δ 1.99 (dd, J= 2.0, 7.0 Hz, 3H), 4.04 (s, 3H), 7.04 (s, ­1H), 7.14 (m, 2H), 7.42 (m, 1H). ^13^CNMR (500 MHz, DMSO-*d*_6_) δ 18.57, 36.38, 127.16, 127.97, 129.26, 143.79, 144.00, 180.80. ESI-TOF-MS: *m*/*z* Calcd. for C_8_H_10_N_2_ONa ([M+Na]^+^) 173.07; Found 173.07

**Compound 3**

**Scheme S7.** Preparation of **3**.

A solution of **1** (202 mg, 1.34 mmol) in 2.0 mL of CH_3_CN/water (7/3) was added to a solution of 2-methyl-1H-indole (**2**, 356 mg, 2.72 mmol) and Cu(NO_3_)_2_・3H_2_O (98.6 mg, 0.334 mmol) in 3.0 mL of CH_3_CN/water (7/3). The resulting mixture was stirred at room temperature for 2 days. CH_3_CN was removed under reduced pressure and diluted with water (30 mL). The aqueous phase was extracted with CH_2_Cl_2_ (30 mL×3), dried over MgSO_4_, and the solvent was removed under reduced pressure. Purification by column chromatography (SiO_2_, hexane/EtOAc (70/30) gave **3** as a brown liquid^7^ (152 mg, 41%). ^1^HNMR (500 MHz, CDCl_3_) δ 1.46 (d, J= Hz, 3H), 2.41 (s, 3H), 3.60 (m, ­2H), 3.78 (m, 1H), 3.86 (s, 3H), 6.93 (s, 1H), 7.04 (m, 2H), 7.09 (m, 1H), 7.22 (m, 1H), 7.66 (d, 1H), 7.70 (s, 1H). ^13^CNMR (500 MHz, DMSO-*d*_6_) δ 12.38, 21.20, 27.15, 36.12, 46.33, 110.28, 115.59, 119. 02, 119.37, 120.67, 126.69, 127.49, 128.94, 130.45, 135.44, 143.42, 192.46. ESI-TOF-MS: *m*/*z* Calcd. for C_17_H_19_N_3_ONa ([M+Na]^+^) 304.14; Found 304.15

**4. Dissociation constants (*K*_d_) of the complexes between monoclonal antibodies (mAbs) and metal complexes**

A 60 µL of solution of mAbs in 20 mM MOPS buffer (pH 6.5) 150 mM NaCl was mixed with a 60 µL of solution of metal complex in various concentrations (10^-7^ M to 10^-3^ M) on a BSA-coated plate. The mixed solutions were incubated at 4 °C overnight and added to a BN(*R*)-BSA coated plate (in the case of mAb R44E1) or a BN(*S*)-BSA coated plate (in the case of mAb S1E11). The plates were incubated at 37 °C for 90 min. After removal of the solutions, the wells were washed twice with 150 µL of washing buffer. Then 100 µL of alkaline phosphatase labeled anti-mouse IgG (1:1000 dilution, in 20 mM PBS) was added and the plates were incubated at 37 °C for 90 min. After removal of the solutions, the wells were washed three times with 150 µL of washing buffer. Then 150 µL of *p*-nitrophenyl phosphate sodium salt in substrate buffer (1.0 mg/mL) was added. Absorbance at 405 nm derived from the product of the enzyme reaction was recorded.

Dissociation constants were determined by Klotz plot:

$$\frac{A_{0}}{A_{0}-A}=1+K_{d}(\frac{1}{c})$$

where *A*_0_ and *A* indicate absorbance at 405 nm in the absence or presence of competitive molecules, respectively. The character *c* shows the concentration of competitive molecules. *K*_d_ represents the dissociation constant.

**Figure S1.** Competitive ELISA of mAb R44E1 for BIQ-PdCl_2_.

**Figure S2.** Competitive ELISA of mAb R44E1 for BIQ-Pd(OAc)_2_ (a)

and corresponding Klotz plot (b).

**Figure S3.** Competitive ELISA of mAb R44E1 for BIQ-PtCl_2_ (a) and corresponding Klotz plot (b).

**Figure S4.** Competitive ELISA of mAb S1E11 for BIQ-PdCl_2_.

**Figure S5.** Competitive ELISA of mAb S1E11 for BIQ-Pd(OAc)_2_.

**Figure S6.** Competitive ELISA of mAb S1E11 for BIQ-PtCl_2_ (a) and corresponding Klotz plot (b).

**Figure S7.** Competitive ELISA of mAb 2B6 (anti-porphyrin antibody) for BIQ-Cu (a)

and corresponding Klotz plot (b).

**5. Docked structures of mAbs with BN enantiomers**

The docked structure of mAbs with BN enantiomers were constructed.^8^

**Figure S8.** Docked structures of mAb R44E1 with BN (*R*) (a) and mAb S1E11 with BN (*S*) (b).

**6. General Procedure for Friedel-Crafts alkylation reaction**

Catalytic reaction was performed in 150 μL total volume containing 1.0 mM of substrates, 50 μM of BIQ-Cu (5.0%) and 50 μM of mAb (5.0%) in 20 mM MOPS buffer (pH 6.5), 150 mM NaCl. The reaction mixture was incubated at 4 °C for 72 h followed by addition of 2-phenyl quinolone as an internal standard for HPLC analysis. The mixture was extracted with diethyl ether (300 μL×3) and the combined organic layer was dried over Na_2_SO_4_ and evaporated under reduced pressure. The residue was dissolved in hexane and analyzed by chiral HPLC. Yields are determined based on peak area at 275 nm using 2-phenylisoquinoline as an internal standard (IS).

**Figure S9.** Calibration curve for **3**.

**7. Dissociation constants (*K*_d_) of the complexes between mAbs and substrates or product of Friedel-Crafts alkylation reaction**

A 60 µL of solution of mAbs in 20 mM MOPS buffer (pH 6.5) 150 mM NaCl was mixed with a 60 µL of solution of substrate 1, 2, or product 3 of Friedel-Crafts alkylation reaction in various concentrations (10^-7^ M to 10^-3^ M) on a BSA-coated plate. The mixed solutions were incubated at 4 °C overnight and added to a BN(*R*)-BSA coated plate (in the case of mAb R44E1) or a BN(*S*)-BSA coated plate (in the case of mAb S1E11). The plates were incubated at 37 °C for 90 min. After removal of the solutions, the wells were washed twice with 150 µL of washing buffer. Then 100 µL of alkaline phosphatase labeled anti-mouse IgG (1:1000 dilution, in 20 mM PBS) was added and the plates were incubated at 37 °C for 90 min. After removal of the solutions, the wells were washed three times with 150 µL of washing buffer. Then 150 µL of *p*-nitrophenyl phosphate sodium salt in substrate buffer (1.0 mg/mL) was added. Absorbance at 405 nm derived from the product of the enzyme reaction was recorded.

Dissociation constants were determined by Klotz plot:

$$\frac{A_{0}}{A_{0}-A}=1+K_{d}(\frac{1}{c})$$

where *A*_0_ and *A* indicate absorbance at 405 nm in the absence or presence of competitive molecules, respectively. The character *c* shows the concentration of competitive molecules. *K*_d_ represents the dissociation constant.

**Figure S10.** Competitive ELISA of mAb R44E1 for substrate **1**.

**Figure S11.** Competitive ELISA of mAb S1E11 for substrate **1**.

**Figure S12.** Competitive ELISA of mAb R44E1 for substrate **2** (a)

and corresponding Klotz plot (b).

**Figure S13.** Competitive ELISA of mAb S1E11 for substrate **2** (a)

and corresponding Klotz plot (b).

**Figure S14.** Competitive ELISA of mAb R44E1 for compound **3** (a)

and corresponding Klotz plot (b).

**Figure S15.** Competitive ELISA of mAb S1E11 for compound **3**.

**8. NMR spectra**

**Figure S16.** ^1^HNMR spectrum of BIQ (500 MHz, CDCl_3_).

**Figure S17.** ^13^C NMR spectrum of BIQ (125 MHz, CDCl_3_).

**Figure S18.** ^1^HNMR spectrum of BIQ-PdCl_2_ (500 MHz, DMSO-*d*_6_).

**Figure 19.** ^13^CNMR spectrum of BIQ-PdCl_2_ (125 MHz, DMSO-*d*_6_).

**Figure S20.** ^1^HNMR spectrum of BIQ-Pd(OAc)_2_ (500 MHz, DMSO-*d*_6_).

**Figure S21.** ^13^CNMR spectrum of BIQ-Pd(OAc)_2_ (125 MHz, DMSO-*d*_6_).

**Figure S22.** ^1^HNMR spectrum of **1** (500 MHz, CDCl_3_).

**Figure S23.** ^13^CNMR spectrum of **1** (125 MHz, CDCl_3_).

**Figure S24.** ^1^HNMR spectrum of **3** (500 MHz, CDCl_3_).

**Figure S25.** ^13^CNMR spectrum of **3** (125 MHz, CDCl_3_).

**9. Chiral HPLC charts**

　(−) and (+) isomers of **3** are defined based on the HPLC analysis with UV and CD detectors.

**Figure S26.** Chiral HPLC charts of (*rac*)**-3** detected with UV (a) or CD (b) detector.

**Figure S27.** Chiral HPLC charts of the products in the Friedel-Crafts reactions catalyzed by mAb S1E11 + BIQ-Cu (a), mAb R44E1 + BIQ-Cu (b) BIQ-Cu (c), 2B6 + BIQ-Cu (d), and BSA + BIQ-Cu (e).

**10. References**

1. Iyoda, M., Otsuka, H., Sato, K., Nisato, N. & Oda, M. Homocoupling of Aryl Halides Using Nickel(II) Complex and Zinc in the Presence of Et_4_NI. An Efficient Method for the Synthesis of Biaryls and Bipyridines. *Bull. Chem. Soc. Jpn.* **63**, 80–87 (1990).

2. Navarro, M. *et al.* Design of copper DNA intercalators with leishmanicidal activity. *J. Biol. Inorg. Chem.* **8**, 401–8 (2003).

3. Khrushcheva, N. S., Bulygina, L. A., Starikova, Z. A. & Sokolov, V. I. Synthesis, structure, and catalytic activity of complexes of 1,1′-bisisoquinoline with PdCl_2_ and NiCl_2_. *Russ. Chem. Bull.* **63**, 883–889 (2014).

4. Bulygina, L. A., Khrushcheva, N. S., Sokolov, V. I. & Khodak, A. A. Synthesis and catalytic activity of a complex of 1,1´-bis-isoquinoline *N,N´*-dioxide with PdCl_2_. *Russ. Chem. Bull.* **64**, 429–431 (2015).

5. Cheng, L.-K., Yeung, K.-S., Che, C.-M., Cheng, M.-C. & Wang, Y. X-ray structure and spectroscopic properties of platinum(II) complexes of 1,1′-biisoquinoline. *Polyhedron* **12**, 1201–1207 (1993).

6. Duchemin, N. *et al.* Expanding biohybrid-mediated asymmetric catalysis into the realm of RNA. *Chem. Commun.* **52**, 8604–8607 (2016).

7. Bos, J., Browne, W. R., Driessen, A. J. M. M. & Roelfes, G. Supramolecular Assembly of Artificial Metalloenzymes Based on the Dimeric Protein LmrR as Promiscuous Scaffold. *J. Am. Chem. Soc.* **137**, 9796–9799 (2015).

8. Adachi, T., Harada, A. & Yamaguchi, H. Development of Atroposelective Antibodies by Immunization with a Racemic Mixture of Binaphthyl Derivatives. *Bull. Chem. Soc. Jpn.* **92**, 1462–1466 (2019).
